# Perception and experiences of senior doctors involved in clincal teaching for Oman’s medical education sector

**DOI:** 10.1186/s12909-025-07904-2

**Published:** 2025-10-17

**Authors:** Laila Moosa Al-Zidjali, Kuang-Hsu Chiang, Hamish Macleod

**Affiliations:** 1 Oman College of Health Sciences, Muscat, Oman; 2 Ministry of Health, Muscat, Oman; 3 University of Edinburgh, Edinburgh, United Kingdom

**Keywords:** Medical doctors’ professional development, Medical policies, Clinical training teaching, Senior and junior medical doctors, Critical realism in medical contexts, Oman medical education, Hospital working environment, Teacher identity teaching experience, Medical doctor mentoring experience

## Abstract

**Background:**

This qualitative study aims to explore senior doctors’ experiences and perceptions of their teaching roles in clinical settings at two hospitals, the Sultan Qaboos University Hospital (SQUH) and Royal Hospital of the Ministry of Health (RH), in the Sultanate of Oman.

**Methods:**

In order to obtain an understanding of doctors’ clinical teaching experiences, this qualitative study uses critical realism as its methodology. In-depth semi-structured interviews were conducted with twenty-seven senior doctors at SQUH and RH. The three-layered realities of critical realism—the Empirical, the Actual and the Real, help generate rich findings and insightful analyses.

**Results:**

The study shows that in the empirical domain, senior doctors perceived their educational roles in a wide spectrum from job titles to the relational nature of their teaching. It was also found that RH doctors felt their teaching roles are less valued than SQUH doctors due to their perception that the teaching mission of their hospital is not formally recognised. Interestingly in the actual domain, there is a lack of clear definition for medical teachers in policies. To explore this further, it is found that in the real domain, although dual missions of Omani hospitals, teaching and health care, are confirmed by Omani authorities, there is seen to be a neglect of educational roles at RH. It was also discovered that professional development offered by the Medical Education Unit (currently known as Medical Education and Informatics Department) for medical teachers is limited due to its exclusion of junior doctors, who do a good amount of clinical teaching. Further, it was perceived as a missed opportunity for the Ministry of Health to recognise medical teachers by not including teaching experience in the promotion criteria for all ranks of seniority.

**Conclusions:**

This qualitative study offers an in-depth understanding of senior doctors’ experiences of clinical teaching at two hospitals in Oman. The lack of clear policies and formal recognition for doctors’ teaching roles, no protected time for teaching, and differential teaching rewards have made RH doctors feel their roles as medical teachers unvalued compared to SQUH doctors. For the Omani government, hospital doctors in Oman would benefit from a positive culture which values medical teaching, gives a sense of belonging for medical teachers in hospitals, offers clear definitions for their teaching roles in policies, and extends the professional development to junior doctors. The study is of value for future research investigating the balance of teaching with other duties of a doctor, and from different perspectives; for example, junior doctors, medical students and other health professionals.

**Supplementary Information:**

The online version contains supplementary material available at 10.1186/s12909-025-07904-2.

## Background

This qualitative research aims to gain an in-depth understanding of how senior doctors perceive and experience their teaching roles within clinical settings in the Sultanate of Oman through the lens of a critical realism framework. Alternative interpretivist and constructivist approaches were considered; however, critical realism was selected for its capacity to explore both observable practices and the institutional and cultural structures that influence clinical teaching roles. At the same time, critical realism presents challenges for researchers unfamiliar with its philosophical underpinnings, as its layered ontological and epistemological concepts can be complex to operationalise in empirical research.

Twenty-seven (27) senior doctors with a minimum of five years of clinical experience from two prominent hospitals, Sultan Qaboos University Hospital (SQUH) and Royal Hospital (RH), participated. SQUH is an affiliated hospital to the Sultan Qaboos University (SQU) and RH is a Ministry of Health affiliated hospital. By investigating these doctors’ perceptions, this study seeks to uncover the underlying mechanisms and structures that shape their teaching experiences.

Teaching, in this study, refers to a broad range of educational activities, from structured teaching of postgraduate and undergraduate students to mentoring or role modelling for colleagues in clinical settings. A significant body of research over recent decades has explored professional development for teaching across various fields, including medicine, nursing, allied health, and vocational training [17, 40, 44, 46, 65, 74, 76]. These studies highlight the evolving nature of teaching and the importance of developing teachers' skills in professional settings.

In some of these settings, such as medicine, teaching and learning have recently become more formalised in nature. For example, it is seen that the curriculum has become structured based on a set of competencies prescribed by medical schools or postgraduate medical training institutions [27, 30, 75]. Some institutions support clinical doctors’ teaching by providing them with guidelines, frameworks and training opportunities [14, 18, 29, 34, 38, 41, 53, 64, 67, 68]. However, some medical teachers continue to face challenges when teaching in clinical settings [9, 15, 69]. Some studies found these challenges facing medical teachers are possibly due to their perceptions of teaching, their heavy workloads with various roles, past teaching experiences and/or their personal lives [8, 19, 20, 43, 57, 66, 73].

### Professional development for medical Teachers in Oman

There are currently 56 government Hospitals in Muscat and the other governorates, with over 6,033 beds and numerous health centres [49]. Professional development for medical doctors’ teaching is not new in Oman. The national programme of professional development for doctors was first agreed upon in 2000 at the Second GCC Conference at King Faisal University in the Kingdom of Saudi Arabia, and was subsequently implemented in 2002 at the Third Gulf Cooperation Council (GCC) Conference of the Faculties of Medicine and Medical Education in Muscat, Oman [33]. To make doctors aware of the importance of teaching, learning and assessment in both classroom and clinical settings, this programme was then developed into the Certificate Course in Health Professions Education (CHPE) by Sultan Qaboos University College of Medicine and Health Sciences (SQU-CoMHS). Over 280 faculty and affiliated faculty have attended these programmes between 2004 and 2015. Oman Medical Speciality Board (OMSB) was then set up in 2006 to develop postgraduate medical speciality education. It sets the professional and educational standards for the training and certification of medical and health professionals in Oman [54].

Although major hospitals such as SQUH and RH offer their own professional development on teaching for doctors who are involved in teaching, most of these activities are ad hoc. No comprehensive orientation is available for new doctors to teach in clinical settings, and little attention has been paid to other health team members, such as nurses, who play an important role in the day-to-day teaching of medical students, health-profession students or residents. More importantly, no one has asked doctors or affiliated health professionals about their teaching needs except through short post-workshop surveys. This contextual understanding is informed by the first author’s professional role within the Ministry of Health, which provided first-hand insight into clinical teaching practices and faculty development activities in major hospitals, and was also examined in the author’s doctoral research [4].

It is in this context that the present study explores how senior doctors experience their teaching roles in clinical settings and what this role means to them in Oman. This study aims to offer critical insights into the perceptions and experiences of senior doctors as teachers within two Omani hospitals with a hope to provide a foundation for policy development and future research.

## Research methods

Data were collected in 2016 using semi-structured interviews with 27 senior doctors at two governmental teaching hospitals in Oman, the Sultan Qaboos University Hospital (SQUH) and the Royal Hospital (RH) (see Supplementary File 1). Critical realism was employed as the overarching methodology to explore the complex, multi-layered experiences of doctors in their educational roles, allowing for a nuanced analysis of both observable and underlying factors influencing their teaching.

### Critical realism as methodology

Although critical realism is widely applied in social sciences research, its use in medical research remains relatively new. Critical realism, due to its unique view of having stratified layers of reality, has been increasingly recognised as a sound tool for obtaining profound understanding of the subject under study [28, 31, 47].

Critical realism was first introduced by Bhaskar in the 1970s and further developed by other scholars such as Margaret Archer, Tony Lawson, Andrew Sayer, Andrew Collier and Alan Norrie [6, 13, 26, 28, 58, 62]. Different from realism, which argues that the world exists independently of social actors, critical realism posits that our knowledge of reality is fallible. This fallibility arises from the fact that it is socially constructed, facilitated by our perceptions and interpretations, therefore, any claims made about reality must be questioned and critiqued to reach the best understanding [5], para. 177,[23, 39, 63].

According to Bhaskar, there are three different domains of reality, namely the empirical, the actual and the real [10, 11], p. 2,[28], pp. 20–21). These three domains are interrelated and overlap. The first domain, the empirical, is made up of the observables or experiences such as outcomes, or phenomena. It is a domain that positivists focus on and have been criticized for, as they are concerned with regularities of phenomena, reducing reality to what is observable and seeking to find “law-like connections” [7, 10], pp. 139–146,[28, 35]. The second domain, the actual, is where events and behaviours take place, independent of observation. [10], p. 16,[28], p. 20).

The third domain, the real, is of particular relevance here. The real is independent of our thought, awareness and even our existence as human actors [10], p. 16,[28], p. 20). Bhaskar described this domain as something with various structures (or objects) which can brings about changes, phenomena or events in the actual domain and then experienced or observed in the empirical domain [11], pp. 35–49). Bhaskar described this domain as consisting of various structures (or objects) and generative mechanisms that have the potential to bring about changes, phenomena, or events in the actual domain, which can then be experienced or observed in the empirical domain (ibid.). Critical realists state that these structures and mechanisms are real, even if they are not tangible or visible, as they can cause events and produce “tendencies,” and it is this that we seek to understand and explain [28], p. 55,[36].

**Fig. 1:**
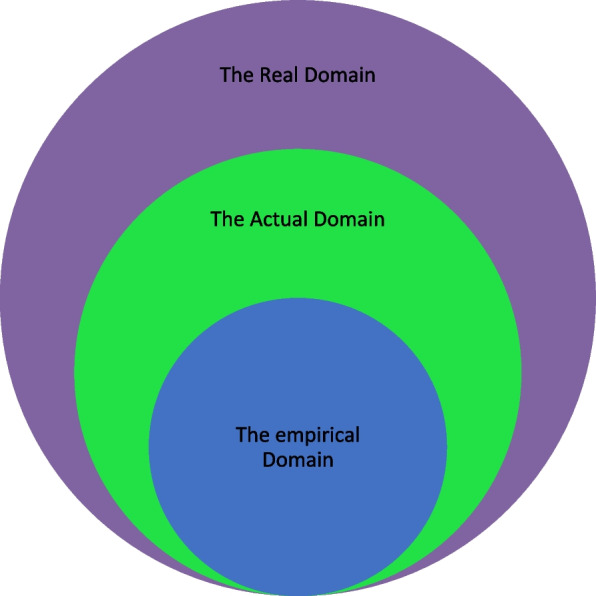
Bhaskar’s Stratified Domains

**Fig. 2:**
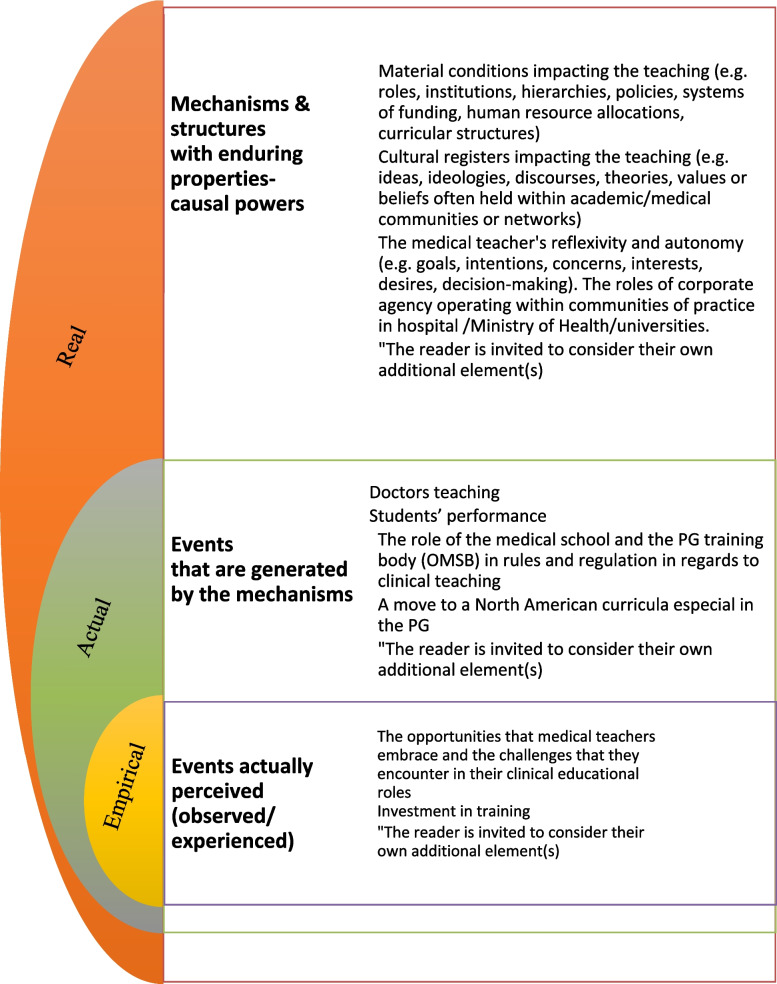
Bhaskar’s Three Domains

Bhaskar’s stratified domains are briefly presented in Fig. 1. The emergence of events in the actual domain and the experiences and observations in the empirical will help to illuminate the deeply rooted mechanisms or structures in the real domain, which are often not easily observed. In this study, teaching experiences of medical doctors’ are treated as the empirical; relevant rules and regulations are seen as events in the actual domain, and finally the socio-cultural structures of the medical teaching system are treated as as the real domain (Fig. 2).

A critical realist perspective guided this study, enabling us to explore not only what senior doctors do as teachers, but also why they do it. This layered view of reality helped uncover structural and contextual mechanisms shaping teaching in Omani hospitals. While realist methodologies are increasing recognised in health professions education globally (Ellaway, Kehoe & Illing, 2020), this is, to our knowledge, the first application of such an approach in Middle Eastern medical education contexts. This study, therefore, contributes to the growing discussion of critical realism’s practical use in medical education and highlights its value in uncovering the often-unseen influences on teaching roles in underexplored contexts.

### Research design

This study collected its data through interviews, supplemented by archival data such as relevant policies and regulatory documents. In-depth open-ended interviews were conducted with twenty-seven senior doctors teaching in clinical settings at Sultan Qaboos University Hospital (SQUH) and Royal Hospital (RH). These two Hospitals were selected due to their significance in medical education and their diverse medical specialties, which provided a rich context for understanding teaching practices. They are drawn from six national hospitals in the Muscat Governorate in Oman. SQUH is a purpose-built university teaching hospital specifically designed for education. It has 700 beds and a staff of 3051 [71]. The RH is a Ministry of Health administrated Hospital with 1241 beds and 3629 staff (Royal [60]—(accessed 11 August 2025).

Criterion sampling was first applied to select participants who met the prespecified criteria (Table 1). These criteria included: (1) participants had to be practising physicians at SQUH or RH; (2) they had at least 5 years of experience teaching medical students and/or trainee doctors at different levels of their training, and (3) they were recognised as a medical teacher by either Sultan Qaboos University College of Medicine and Health Sciences (SQU-CoMHS) or Oman Medical Specialty Board (OMSB), or both [21], p. 143,[24], p. 224).

**Table 1:** Criteria for selection of participants

Sampling Type	Criteria
Inclusion – Criterion Sampling	– Practicing physician at either SQUH or RH – Teaching medical students and/or trainee doctors at various levels for at least 5 years – Recognized/certified as teachers by either SQU-CoMHS or OMSB, or both
Inclusion – Purposive Sampling	– Gender – Specialty (e.g., surgery, cardiology) – Clinical setting (e.g., intensive care unit, outpatient clinic) – Years of clinical experience – Years of teaching experience – Formal training in medical education (e.g., certificate, diploma, or master's degree) – Study and training backgrounds (including overseas-trained doctors), to explore whether these factors influenced their teaching practices
Exclusion Criterion	Any physician with no teaching board certification^a^

^a^Board certification indicated the education level achieved by physicians beyond their undergraduate certification as medical doctors and as a minimal standard of required core competencies in their chosen specialty

Subsequently purposive sampling was adopted to select participants, who could offer diverse and novel perspectives on the experiences of clinical teaching [21], p. 104,[24], p. 262). Additional Criteria included medical doctors’ gender, medical specialty (e.g., surgery, cardiology etc.), clinical settings (e.g. intensive care unit versus outpatient clinic), clinical and teaching experiences, formal training in medical education (e.g. certificate, diploma, or master's degrees), and overseas training, which can offer different strategies for improving teaching practices.

The semi-structured interview guide (see Supplementary File 1) was informed by the study’s research questions and sub-questions, which were themselves derived from the overarching research aim. The guide consisted of eight open-ended questions that explored participants’ teaching roles, responsibilities, and how these may have changed over time. It was piloted with two senior doctors from SQUH under conditions similar to the main study. No changes were required following the pilot, and both participants were included in the final sample. This process confirmed the guide’s appropriateness and contributed to preparation for the interviews. The interview protocol was also reviewed by the doctoral supervisory team. Although the questions were not framed using critical realist terminology, the interview guide was shaped by the ontological and epistemological assumptions of the critical realist approach.

Purposive sampling also considered doctors with diverse study or training backgrounds, including those who had received their undergraduate or postgraduate medical education overseas. The inclusion of these participants was intended to explore whether exposure to different cultural and educational systems shaped their perspectives and approaches to clinical teaching. This strategy helped ensure a diverse sample that reflected variation in teaching perspectives across specialties, institutions, studying and training backgrounds, and experience levels.

To determine data saturation, the principle of thematic redundancy was adopted—defined as the point at which no new information, codes, themes, or insights were emerging from the interviews [24], p. 262,[25], p. 270,[45], p. 95,[61]. Initially, a manageable number of participants were chosen from both institutions to be interviewed. Saturation was assessed through reflective memoing during interviews and preliminary thematic analysis. At SQUH, saturation was reached after 11 participant interviews. On the other hand, at RH, more participants were recruited to ensure sufficient thematic coverage, resulting in twenty-seven participants—11 from SQUH and 16 from RH (Table 2). During the interview process, eight main semi-structured questions were explored, with slight variation in the order depending on the participants’ answers, which led on to include issues to be raised by later questions. NVivo 12 software was used to analyse interview data.

**Table 2:** Demographics of participants

Category	Subcategory	SQUH	RH	Total
Gender	Female	1	4	5
	Male	10	12	22
Clinical Experience	< 5 years	0	0	0
	5–10 years	1	0	1
	11–15 years	2	5	7
	> 15 years	8	11	15
Teaching Experiences	< 5 years	0	0	0
	5–10 years	5	8	13
	11–15 years	3	4	7
	> 15 years	3	4	7
Specialty	Medical	3	4	7
	Surgical	2	3	5
	Anaesthesia & Intensive Care Medicine	0	2	2
	Emergency Medicine	3	1	4
	Paediatric	3	4	7
	Radiology	0	2	2
Studying & training country/region (Undergraduate medical study)	Oman	8	12	20
	India & Pakistan	2	2	4
	Egypt	1	0	1
	Canada	0	0	0
	UK & EU Countries	0	2	2
	Australia	0	0	0
Studying & training country/region (Postgraduate residency training)	Oman	1	3	4
	India & Pakistan	2	2	4
	Egypt	1	0	1
	Canada	6	6	12
	UK & EU Countries	1	4	5
	Australia	0	1	1

### Research ethics

This qualitative non-clinical study adhered to strict ethical guidelines. While the study was based in the Medical School of the University of Edinburgh, the study received scrutiny and approval from the Ethics Committee of the Moray House School of Education and Sport with which medical educational research at Edinburgh was affiliated. Additionally, ethical clearance was obtained from the Research and Ethical Committee at the Ministry of Health in Oman and the Sultan Qaboos University Medical Research and Ethics Committee, where the data collection took place. Throughout the study, participant anonymity and confidentiality were strictly maintained. Informed consent was obtained from all participants, including explicit agreement that interviews could be audio-recorded, transcribed, and used in anonymised form for the doctoral thesis, academic publications, and conference presentations. These measures ensured the protection of participants' rights and the ethical integrity of the research process.

## Findings of Doctor’s teaching experience

This section firstly presents three key findings: perceptions of their teaching roles, the importance of the teaching role recognition, and the absence of clear educational role descriptions, where the first two are in empirical domain and the third in the actual.

## The empirical

### Perceptions of teaching roles

Most participants, despite having over five years of clinical teaching experience, initially described their educational roles in terms of their formal job titles and institutional responsibilities, such as program director, department head, teacher, trainer, physician, or consultant. Roles such as scientific committee chair, committee member, and supervisor were also mentioned. However, few participants initially identified the relational aspects of their roles, such as being a mentor, facilitator, or role model for peers and students. These relational elements often seem to be overlooked or not formally recognized as part of their teaching role.

Upon further exploration, participants described their roles as medical teachers with greater nuance, adding depth and meaning to their initial descriptions. A key dimension highlighted was the importance of relationships with colleagues, students, and junior doctors. They identified themselves as mentors to junior doctors and role models for both students and colleagues, treating students and junior doctors as future professional peers. Participants have shown altruistic behaviours, such as being kind and going out of their ways to teach and include them in the clinical decision-making process. For example, S09 at SQUH who was actively involved in junior doctors’ training shared how he relates to residents as colleagues, working closely with them in daily clinical tasks and maximising learning opportunities in both classroom and clinical settings.

[I am] heavily involved [with the postgraduate program]... so we design their rotations, the curriculum and their didactic teaching, so this is in terms of planning. In terms of implementation, we interact with the residents on a daily basis... we review the cases with them and do the teaching along with the clinical work. Also, with that, we do didactic sessions.

(S09, SQUH)

Several participants from both hospitals saw themselves as catalysts for change within their teaching environments. When recognizing a need for improvement, they would begin by altering their own behaviour, with the hope that colleagues and students would emulate these changes. Such changes were noted in areas like revising specialty curricula, adopting new teaching methods, and reshaping relationships with students and colleagues. Additionally, participants noted that their teaching roles evolved as they progressed in their medical careers, particularly through leadership roles assigned by the rota system.

A notable distinction emerged between doctors at SQUH and RH in terms of how they perceived their roles as medical teachers. Participants from SQUH often identified themselves as teachers, viewing teaching as an integral part of their professional role. In contrast, doctors at RH were less likely to explicitly identify teaching as part of their role, reflecting differences in institutional expectations and cultural contexts.

At SQUH, participants with educational administrative duties such as a director of educational programmes viewed teaching undergraduates and postgraduates as an inherent aspect of their role, requiring no further prompting. They clearly declared that they are teachers. For example, S05 (SQUH) spoke with joy and enthusiasm about his role in teaching clinical skills and other essential competencies in a clinical setting.

My current roles, obviously I teach... In the clinical years, it’s more of teaching at the bedside as well as in seminars. I teach not only [my specialty], but we teach medicine, we teach things like quality management, patient safety and professionalism, et cetera.

(S05, SQUH)

Similarly, S04 (SQUH) saw his clinical teaching role as twofold: acting as an adviser who provided direction and guidance to medical students, while also serving as an organiser and a facilitator for junior doctors.

To start with, my educational role for the undergraduate is, I usually give either didactic lectures or clinical bedside teaching. For the clinical bedside teaching, my role is to guide the students.... For postgraduate teaching, usually, we start the education with clinical bedside teaching.... we will go into a group discussion, and we would only be like a facilitator for them.

(S04, SQUH)

S11 (SQUH) perceived the training of undergraduates and postgraduates as an integral part of his professional role and responsibilities. Many SQUH doctors pointed out that teaching is expected of them as part of their roles at a university hospital like SQUH. S08 (SQUH) emphasised this by stating, “actually, in the college, we don’t have like specific, educator or teacher roles. I think it’s inherited, also part of like, you are in the college, you are a teacher, so you have to be a teacher” (S08, SQUH). For some SQUH doctors, responsibilities assigned by OMSB was considers as an added role, either it’s administrative, supervising, teaching or evaluating the junior residents in training.

S11 (SQUH) highlighted the dual responsibilities he had as both a clinician and an educational programme director. He described his routine of balancing clinical duties and supervising residents:

This is a University Hospital training site where we receive undergraduate and postgraduate. And in addition, I have [significant administrative oversight] for the [specialty residency] programmed of OMSB. So I supervise and interact with the resident both as an administrator and at work. So I do clinical on average two to three clinical days per week, okay. And most of the time, I have trainees working under me during the shift. So during the shift, I will supervise their cases, give them teaching, you know. And also, maybe we will go beyond that to discuss some theory, and I’ll give them evaluation as well.

(S11, SQHU)

In contrast, most of the RH participants, even those officially recognised by the OMSB as “official trainers”, did not explicitly identify teaching as part of their role. Instead, they referred to their educational programme director role as their primary educational role, only mentioning clinical teaching when reflecting on past educational roles, challenges or opportunities. They tended to use the word “trainer” used by OMSB, rather than “teacher”. For example, R06 from RH explained his longstanding involvement in the organization, continuing his role as a trainer while serving on various committees:

I was one of the people [with longstanding organisational involvement] and now I’m... continue my role as a trainer, as well as a member of the Scientific Committee. And within the Scientific Committee of the Residency Program, there are subcommittees. I am a member of the Subcommittee of the Examination, as well as the Resident Progress Subcommittee.

(R06, RH)

Similarly, R02 (RH) emphasized his official trainer status within the residency program:

I am an official trainer in [my specialty] residency program for the Oman Medical Specialty Board, and I have been doing this for the last three years. And previously, over, like, six years I was then doing clinical teaching as part of my training, being a senior resident to teach the juniors.

(R02, RH)

Despite the differences in roles, all participants at SQUH and RH emphasized the importance of building relationships with their students. They appreciated the opportunity to learn alongside their students and enjoyed the interactive aspect of teaching. This approach reflected their own experiences as students, where they often benefited from close interactions with their mentors. Participants viewed the clinical setting primarily as a space for students to develop their clinical skills rather than merely a venue for assessing their knowledge. As R09 expressed, “I like direct interaction rather than giving lectures [to the students and the residents]” (R09, RH).

### Importance of recognising teaching

Participants at RH expressed that their teaching roles were not adequately recognized by their hospital. They highlighted a disconnect between the practical reality of their teaching responsibilities and the formal recognition on paper. Participants pointed out that although their institution (RH) functions as a teaching hospital in practice, this is not officially acknowledged. They believed this led to a lack of support from the Ministry of Health. They believed if their hospital were formally recognized as a teaching hospital, their teaching experience would improve. The perceived lack of recognition, they argued, resulted in a corresponding lack of institutional support.

R02 (RH) believed that their hospital’s fundamental mission was to provide quality healthcare, a goal that has remained unchanged. But in reality, RH has also become a teaching and training site for medical students in Oman and residents from OMSB, as well as nursing and allied health students, as well as those from overseas. Participants at RH therefore felt that they are not officially recognised as teachers, even though they actively engage in teaching.

[This hospital] started being like service, that was in ’86, it never aimed to be as a teaching hospital, and it’s continued from that time. So, it is definitely a teaching hospital in practice, but on paper, it’s not considered to be a teaching hospital.

(R02, RH)

Further, R15 (RH) emphasised that RH doctors are not recognized as medical teachers by SQU-CoMHS, which resulted in a lack of access to essential teaching resources, such as the electronic library. In comparison, affiliated doctors at SQUH have full access to these resources at Sultan Qaboos University. He said, “You’re receiving the students [at RH], you’re teaching them, you’re getting an allowance for that, once a year, but you’re not given access to their electronic library [at Medical College]” (R15, RH).

A further reason for the perceived lack of recognition is disparity in teaching rewards between the two hospitals. S08 (SQUH) pointed out that all doctors at SQUH receive a monthly teaching allowance, regardless of whether they actually teach.

Like you know, if you talk about the affiliated hospital, they don't get an allowance for teaching. We get an allowance for teaching. But also I think we get an allowance for teaching here, but we have also teachers whom they don't teach.

(S08, SQUH)

In contrast, doctors at RH who are officially recognised as teachers by SQU-CoMHS or OMSB receive only a small financial reward or a yearly teaching bonus, according to R15 and other RH participants. The disparity in rewards may contribute to RH doctors feeling less recognized compared to their SQUH counterparts. Could this difference explain why SQUH doctors are more likely to identify themselves as teachers?

R03 at RH described how the differential in teaching rewards between SQUH and RH, where the former receives an allowance and the latter merely a bonus, affects how RH medical teachers feel inferior and unappreciated in comparison to their SQUH counterparts.

The second [reason] is also the financing for these teachers. It is completely different from the Sultan Qaboos University, Sultan Qaboos University Hospital. For them, it is fixed, and it is as an allowance; for us, it is only just a bonus. And it is a big difference between these [at SQUH] and us, especially the tutor here. They have a sense of... we cannot say inferiority, but we can feel the difference. And usually, we overcome it by saying this is our country and those are our people, so it will help to teach them.... because this discrimination it causes some sort of sensitivity between the clinicians.

(R03, RH)

## The Actual

### Absence of clear roles for medical teachers

Many participants expressed their concern about the lack of clarity surrounding their expected teaching roles. For this purpose, SQU-CoMHS was contacted for relevant documents, but no such documents existed, nor were they available in public domain, such as the SQUH website (accessed 11 August 2025).

On the other hand, OMSB provided two relevant documents: the** “**OMSB Trainer Manual” [55] and “Program standards “P” and training center standards “T” for OMSB residency program Booklet” [55]. On examining the former (ibid., 2014), it was found that the manual is seen to offer comprehensive guidelines for trainers, rules and regulations for scientific committee members, selection criteria for committee members and trainers, trainer responsibilities, performance monitoring, and relevant OMSB policies and procedures. The latter [56] highlighted the trainers’ responsibilities that are obligatory. For example:

6B.5 The Trainers must provide graded supervision appropriate to the competence and experience of the Resident and decide on awarding EPAs to the Residents as deemed appropriate.

[56]

The “OMSB Program and Training Standards” also specifies 15 key responsibilities for trainers, including becoming familiar with rotation objectives, supervising residents, providing continuous feedback, offering procedural opportunities, maintaining a conducive educational environment, and participating in faculty development and academic activities. Trainers are also expected to provide protected teaching time and regularly evaluate residents using OMSB evaluation forms ([56]: 52–53).

The above likely explains why some RH participants felt that being officially recognised as a trainer by OMSB helped clarify their teaching roles (R10 & R02, RH). For example, R04 (RH) found the “Training of Trainers Workshop” offered by OMSB particularly useful for understanding his teaching responsibilities (R04, RH)). Similarly, R02 (RH) perceived that his teaching role becomes more structured after he became recognized as an official OMSB trainer:

I think it was most important when I got this role of, you know, trainer in OMSB, to become more structured with the commitment.... You know how you are doing when you get evaluated. And you know which path you are taking, what are the criteria, what are the objectives, and what I’m doing means. So it’s more structure than the previous year.

(R02, RH)

While joining OMSB and being recognized as official trainers made doctors more aware of certain aspects of their teaching roles, such as learning objectives, assessment and evaluation, for many participants it did not fully address the lack of clarity regarding their broader educational roles. RH participants, in particular, were inconsistent and unsure whether a written document existed that defined their educational roles for undergraduates and postgraduates. R04 (RH) felt that although some initial information was provided when becoming an official medical teacher for teaching the medical students from Sultan Qaboos University, it did not clearly specify the expected teaching role. It was only the document that described the objectives expected of students that was provided. This view was also echoed by other RH participants with R14 expressing that “we just follow the objectives that are written down” (R14, RH). S05 (SQUH) also highlighted that his teaching role was not clearly specified by SQU-CoMHS beyond the objectives set for students.

While RH participants like R04 and R14 focused more on the absence of written role descriptions for both undergraduate and postgraduate teaching, SQUH participants, such as S08 and S10, emphasized that teaching is an implicit expectation within their university roles, though they similarly lacked specific, formalized role definitions.

Actually, in the college, we don’t have like specific, educator or teacher roles. I think it’s inherited, also part of like, you are in the college, you are a teacher, so you have to be a teacher. (S08, SQUH).

OMSB does have that, I don’t recall as specific as OMSB document.... [In] the college we know the goals, overall goals, but I didn’t see those specific point by point role of the trainer.” (S10, SQUH).

SQUH participants tended to express a greater desire for clearly defined boundaries between their various responsibilities, reflecting the complex nature of their working environments.

“Now we’re living in a place or at a time where everybody is asking a piece of you, so knowing the exact roles, the exact responsibilities, and rights, and having that in writing so that you’ve actually got something to refer to.... so tell me what’s your expectations so that I know if I can deliver that or not; that’s very important. (S08, SQUH)”

Moreover, participants emphasised the need for educational institutions to adopt innovative and varied methods of communication with their medical teachers. S05 (SQUH) admitted that “Yes, the curriculum and the written document are there, but written documents are written documents. We read it once, and then we never look at it again” (S05, SQUH). S08 pointed out that written communication serves to build relationships and foster trust between the doctors and the educational institutions. According to R08 (SQUH) and other participants, effective communication would help align goals of all stakeholders, including teachers, hospital administration, and educational institutions. They believed that clear communication would eliminate confusion about their expected educational roles and ensure alignment with the learners’ learning objectives. S08 said:

Now we’re living in a place or at a time where everybody is asking a piece of you, so knowing the exact roles, the exact responsibilities, and rights, and having that in writing so that you’ve actually got something to refer to.... so tell me what’s your expectations so that I know if I can deliver that or not; that’s very important.

(S08, SQUH)

To establish standardised roles for medical teachers nationwide, S02 (SQUH) suggested that medical teachers themselves should develop the criteria and requirements for their roles. He perceived that the absence of a “critical mass” of educators and a lack of policies and guidelines, had led to inconsistent practices. “I think there are no standards now for medical teachers. If you want to talk nationwide, there is no set of standards” (S02, SQUH).

S02 (SQUH) further emphasised the need for better coordination among hospitals and relevant authorities. He pointed out that medical education institutions and healthcare providers lack proper communication and alignment regarding doctors’ educational roles. He believed that those institutions must collaborate more effectively to meet their educational needs, adding, “I think if we harmonise and become one whole, it will be better” (S02, SQUH).

S02 (SQUH) argued that establishing clear teaching roles for medical teachers requires input from all stakeholders. He suggested that doctors themselves should take part in developing standardised criteria for their educational roles. Such standards would enable educators to differentiate not just students at different levels of learning but also their junior colleagues and their levels of learning.

What happens now is that we tend to mix our role. So we can be very harsh to the medical students who are still junior, and we take the role of teaching residents. So we don’t know in our mind and how, for example, to differentiate between a junior and a senior clerkship student and a resident R1 and the senior resident. So this role is not really clear in daily practices.

(S02, SQUH)

S02 (SQUH) and other participants discussed how the lack of clarity about educational roles had affected their ability to differentiate between learners at various levels. This ambiguity added another layer of complexity to their already challenging clinical environments, contributing to feelings of uncertainty in their teaching roles. Despite these challenges, it was their sense of duty they felt toward their students that helped them navigate these difficulties in clinical settings.

## Discussion and interpretation of the real

Critical realists question any claim made about reality, arguing that our knowledge of reality is fallible due to two factors: first, it is constructed by social actors who perceive and interpret the world around them, secondly, and it is constrained by the conceptual, theoretical, and methodological tools through which we investigate it [5], para. 177,[23], paras. 23, 25,[39, 63]. Despite this, critical realists believe that there is a reality out there independent of those social actors' perceptions [5], para. 177,[12], para. 78,[23], para. 62,[39, 42, 63]. In this section, we explore how the findings from the empirical and actual domains can be attributed to structures in the real domain, focusing on the factors that shape medical doctor’s experiences and perceptions of their teaching roles in clinical settings.

### Teaching as one of dual missions for all Omani Hospitals, But RH?

One important structure in the real domain is Omani medical education, which has been evolving since the 1980 s with the establishment of the College of Medicine and Health Sciences at Sultan Qaboos University (SQU-CoMHS) in 1986, the first Omani teaching hospital, Sultan Qaboos University Hospital (SQUH) in 1990, and the Oman Medical Specialty Board (OMSB) in 2006. SQU-CoMHS partnered with SQUH to ensure that Omani hospitals not only provide healthcare but also offer medical education, recognizing teaching and healthcare as dual missions.

Surprisingly, RH doctors did not perceive their hospital as intended to be a teaching institution. This contradicts the hospitals’ dual mission and the original goal of the Oman Project, developed in partnership with the Royal College of Physicians and Surgeons of Glasgow (RCPSG). RCPSG helped the Ministry of Health establish RH with state-of-the-art facilities for postgraduate training in the early 1980s [37]. Former colleagues from the first generation of doctors at RH confirmed that its teaching mission was to train medical students, a goal reflected in RH’s current vision which is to train both undergraduate and postgraduate medical and allied health students [50], accessed 11 August 2025).

The Ministry of Health [50] (accessed 11 August 2025) website clearly confirms that RH is a teaching hospital, not only for undergraduate medical students but also for the postgraduate residency program of OMSB. It also serves as a centre for the membership examinations for the Medical Royal Colleges of UK and Ireland and provides clinical training for the nursing and health students from affiliated institutions.. The Ministry of Health [50] stated:

The Royal Hospital is a major teaching hospital for the MD course conducted by the Sultan Qaboos University. It also serves as the main training facility for the postgraduate Residency Program of the Oman Medical Specialty Board in Medicine, Surgery, Obstetrics & Gynaecology, Child Health and Laboratory Medicine. Further, the Royal Hospital is recognized by the Royal Colleges of UK and Ireland as an official centre for the membership examinations in Medicine, Paediatrics and Surgery. Nursing and paramedical students of the Nursing Institutes in the capital area, the Institute of Health Sciences, and the Sultan Qaboos University are also provided clinical training at the Royal Hospital.

[50] para. 6-accessed 11 August 2025)

SQU-CoMHS also identifies RH as a teaching hospital [22], pp. XVII & 55). It recognized those who teach medical students as affiliated teachers:

The [Royal Hospital] is a teaching tertiary Hospital with 623 beds and 265 senior specialists and senior/consultants (70 affiliated teachers) and 1,298 nurses. Like SQUH, it receives referral cases from all over Oman. In the RH, the students rotate in medicine, child health, paediatric, surgery, Ob/Gyn, surgery, accident & emergency, radiology and anaesthesia departments.

[22], p. 55) (CoMHS, 2013: p.55)

The Ministry of Health's Code of Ethics for Doctors (2007) states that, in addition to their clinical duties, doctors are responsible for educating others, including patients, colleagues, and students in medical, nursing, and allied health. The code emphasizes that, as educators, physicians must:

- Spread health education among patients, family and the community
- Develop skills, attitudes and practices of a competent teacher.
- Teach and supervise adequately your junior colleagues, medical, nursing and paramedical students.
- Be honest and objective when assessing the performance of those whom you have supervised or trained.

[48], p. 13)

It is therefore safe to say that RH is a teaching hospital. However, the question remains: why do senior doctors at RH continue to demand official recognition of their teaching roles? What leads RH participants to feel that their hospital is not truly a teaching institution, and why does this discrepancy exist?

Two possible reasons explain this discrepancy. First, this is possibly because RH is administered by the Ministry of Health (MoH), which is not an educational institution. In Oman, it is only educational institutions such as the College of Medicine and Health Sciences of Sultan Qaboos University (SQU-CoMHS) or OMSB, not the MoH or RH, that can certify doctors as medical teachers. Therefore, doctors at RH rely on sources or recognition at SQU-CoMHS or OMSB for their professional development as medical teachers. There is no real recognition of teaching at the institutional level within RH or by the Ministry of Health, despite existing policies. This is reflected in the differential treatment of doctors with teaching duties at RH with those at SQUH. There is a lack of protected teaching time, inadequate facilities and equipment, and insufficient rewards for their teaching efforts for doctors at RH. These factors likely contribute to the feeling that their teaching roles are undervalued by RH and Ministry of Health.

Second, a sense of belonging to the institution plays a significant role. Senior doctors at SQUH perceive their hospital as a teaching institution, where teaching is an integral part of their clinical and other responsibilities. Many participants from SQUH view their roles as both clinical and educational, as they teach undergraduate and postgraduate students. S08 remarked, “I teach undergrads as part of the faculty in the College of Medicine… And also, I teach postgrads like OMSB residents” (S08, SQUH). S09 added, “I work in Sultan Qaboos University, which is a teaching hospital for both undergraduate and postgraduate” (S09, SQUH) while S11 confirmed, “this is a University Hospital training site where we receive undergraduate and postgraduate” (S11, SQUH).

In contrast, RH doctors lacked this sense of belonging. Medical teachers at RH often felt undervalued and disconnected from their institution. This aligns with broader challenges reported in the Gulf region, where institutional support for clinical teaching is uneven, particularly outside university-affiliated hospitals [3]. Many believed that teaching at RH would become unsustainable over time and that the quality of teaching would deteriorate unless significant changes were made to better support RH’s medical teachers.

### Limitations of professional development for medical teachers

Another important structure is the Medical Education Unit (MEU (currently known as Medical Education and Informatics Department)) established in 2000 at the College of Medicine and Health Sciences of Sultan Qaboos University (SQU-CoMHS), which later became the Medical Education and Informatics Department. MEU offers professional development opportunities, not only for those at SQU but also for those at other affiliated teaching hospitals, and helps foster a medical education culture not only for the doctors at SOUH but also for those at RH too. However, MEU’s impact is limited. It prioritises SQUH doctors over those at RH, and its high entry requirement—requiring all applicants to be senior doctors and prior attendance at SQU-CoMHS training -creates barriers for junior doctors and other medical teachers. This produces a false impression that ‘unadmitted doctors’ are not qualified and not supposed to teach.

Although SQU-CoMHS mandates that those who teach medical students must be senior specialists or consultants, in reality most senior doctors are too busy to teach. As a result, teaching responsibilities are often delegated to junior doctors. R14 and R05 at RH explained the challenge of balancing teaching with their administrative and clinical duties, which often leads to passing these tasks to subordinates.

Of course, when you become at a level where your responsibilities are, sort of, becoming more and expanding, then it is not easy to keep up with your momentum, giving the sessions. So, you tend to delegate to other junior doctors or junior colleagues who can replace you.

(R14, RH)

Most available studies on faculty development in the Middle East—such as Algahtani et al. [3], which surveyed academic staff in Saudi medical colleges—focus on institutional settings within universities. These studies do not directly explore the experiences of hospital-based clinical teachers, particularly those outside of university-affiliated hospitals. Consequently, they may overlook the unique professional development needs of doctors who assume teaching roles in clinical settings.

While it is not unusual for junior doctors or other professionals for example nurses, or even patients to teach medical students or residents [16, 59, 70], it is surprising that junior doctors who actually take on teaching roles are not eligible for professional development of teaching offered by MEU. This devalues the teaching contribution made by junior colleagues and sends a negative signal about teaching. R14 and other participants at RH expressed concern about assigning teaching duties to those junior doctors who haven’t received any training as a teacher.

Addressing these gaps requires structured evaluation mechanisms tailored for clinical teaching environments.

Although tools such as the Maastricht Clinical Teaching Questionnaire (MCTQ) have been validated in a Middle Eastern context [2], their routine use for evaluating or developing clinical teachers in hospital-based settings—particularly in Oman—has not been widely reported in the literature. Such tools, if systematically adopted, could help institutions recognise and support clinical educators, especially in non-university teaching hospitals.

Despite a regional literature search, no comparable studies were found focusing on the clinical teaching roles of senior doctors in hospital-based settings in the Middle East. This highlights the novelty and contribution of the current study.

### Missed opportunity to recognise clinical teaching?

A mismatch exists in how clinical teaching is recognised in promotion criteria for doctors. Since Oman Medical Speciality Board (OMSB) was established in 2006 aiming to “achieve excellence in postgraduate medical education, training, assessment and accreditation” in order to “educate the next generation of leaders in medicine” ([54], p. 3). An important medical bylaw passed in 2014 highlighting the value of teaching in a clinical setting by specifying teaching is a criterion for promotion to senior consultant: doctors “should be involved in teaching and educational activities at educational, training and health institutions” [51], pp. 87–88).

However, the bylaw does not mention teaching as a criterion for promotion below the rank of senior consultant or once a doctor becomes a senior consultant. As a result, some doctors opt-out from their teaching duties once they are promoted to senior positions. This unfortunate omission is perceived as a ‘missed opportunity’ on the part of the Ministry of Health to promote clinical teaching for all doctors. Both R11 and R12 at RH believe that some doctors opt out of teaching because there are no consequences for not teaching, especially when teaching is perceived as an extra burden. R11 said “I have not been given a dedicated time [for teaching], so it’s understood that I will teach if I have time. If I don’t have time, I’ll not teach. So there is no compulsion on me to teach.” (R11, RH).

### Research limitations

One constraint of this study was about the limited access to senior doctors due to their busy and often unpredictable nature of their clinical and administrative work. Although the administrative support was obtained from the directors of both hospitals, some doctors were unavailable, and few declined or did not respond despite follow-ups attempts due to time constraints or rotation schedules. Where possible, interviews were rescheduled to accommodate participants’ availability.

While there were only 5 female participants out of the overall group of 27 senior doctors, this did not under represent the female voice in this study as only 6% of senior doctors in Oman are female [52].

## Conclusions and recommendations

This study provided important insights into how senior doctors in Oman understand and navigate their teaching roles in clinical settings. Using a critical realist lens, it explored both the observable teaching practices and the deeper institutional and cultural structures shaping clinical teaching.

The findings revealed marked differences between the two hospitals. At both sites, doctors valued the relational aspects of their teaching and regarded it as a meaningful component of their professional role. However, doctors at RH described feeling undervalued and less supported, with fewer opportunities for recognition and development. Unlike their SQUH counterparts, many RH participants did not view teaching as an integral duty, reflecting the lack of protected time, limited financial rewards, and unclear role expectations. These disparities fostered a sense of marginalisation among RH doctors, contrasting with the stronger teaching identity evident at SQUH.

These disparities reflect broader systemic challenges in how clinical teaching is recognised, supported, and structured. Institutional support is inconsistent, access to professional development opportunities is limited—particularly for junior doctors—and teaching is not uniformly acknowledged in promotion pathways. Addressing these issues is essential to strengthening the medical education system in Oman.

Based on these findings, the following recommendations are proposed:

1. Develop National Teaching Standards: Introduce a unified national framework for clinical teaching roles, modelled on standards such as the UK Professional Standards Framework and the Academy of Medical Educators' standards [1, 32, 72].
2. Enhance Institutional Recognition and Support: Ensure that all hospitals, including those outside university systems, formally recognise clinical teaching as part of a doctor’s role. This should include providing protected teaching time, equitable financial rewards, and access to academic resources.
3. Inclusive Faculty Development: Broaden eligibility for teaching-related professional development to include junior doctors and all staff involved in clinical teaching, regardless of seniority or formal institutional affiliation.
4. Support Teaching Identity Formation: Embed teaching skills in undergraduate medical education and recognise teaching contributions during postgraduate training to support the early development of teaching identity. This could help position teaching as a core component of medical professionalism.

Future research should incorporate the perspectives of junior doctors, healthcare professionals, and medical students. It should also examine clinical teaching across different levels—undergraduate, postgraduate, and residency—and explore how doctors balance teaching with clinical and administrative responsibilities. Gender disparities in the Omani medical workforce, as in many countries, further highlight the need to investigate the unique experiences of female doctors involved in teaching.

Creating a culture that values clinical teaching would requires commitment from all stakeholders. Coordinated action between the Ministry of Health, medical schools, and hospital leadership is essential to ensure that all doctors—regardless of hospital or rank—are supported in their teaching roles and identities, thereby strengthening the quality of medical education and patient care in Oman.

## Supplementary Information

Supplementary Material 1

## Data Availability

The datasets generated and analysed during the current study are not publicly available due to confidentiality agreements with participants. However, de-identified excerpts are available from the corresponding author on reasonable request.

## Abbreviations

MEU: Medical Education Unit (currently known as Medical Education and Informatics Department)
RH: Royal Hospital
SQUH: Sultan Qaboos University Hospital
GCC: Gulf Cooperation Council
CHPE: Certificate Course in Health Professions Education
OMSB: Oman Medical Speciality Board
SQU-CoMHS: Sultan Qaboos University College of Medicine and Health Sciences
RCPSG: Royal College of Physicians and Surgeons of Glasgow
