# Supplementary material for: Perception and experiences of senior doctors involved in clincal teaching for Oman’s medical education sector
Source: BMC Med Educ. 2025 Oct 17;25:1446. doi: 10.1186/s12909-025-07904-2 (PMC12535085; doi:10.1186/s12909-025-07904-2)
Supplement: Supplementary file 1 — Supplementary Material 1 [file 12909_2025_7904_MOESM1_ESM.docx]

### Supplementary File 1: Semi-Structured Interview Guide

1. Could you please tell me about your current role as a clinical educator that you have been doing now for X years? Could you please tell me what you do in this role?

**Possible prompts:**

- how long have you been doing this role?
- What is it like to be a medical teacher?
- What do you most enjoy about your clinical educational role?
- What are its challenges- if any? And why?
- What do you think of other medical educators’ roles?

1. Could you please tell me whether you had any thoughts about clinical teaching whilst you were a student or resident?

**Possible prompts:**

- Have you thought much about your clinical teachers? Give real examples.
- Have you had any expectations or worries about doing it right when it becomes your turn to teach? If yes, what and why is that; if no, why not?

1. What preparation, if any, have you had for your clinical educational role?

**Possible prompt:**

- Have you required assistance from anyone to do your role?
- What challenges have you been expecting in this role?
- How have you dealt with such challenges?
- What opportunities have you had?

1. If you have not been prepared, why do you think this might be?
2. If you have been prepared – has anyone helped you with this?

**Possible prompts:**

- Has anyone helped you to be prepared for your clinical educational role?
- Have you talked about your role with anyone?
- Do you think you might change your role in any way?

1. If you have not had any help – would you like some?

**Possible prompts:**

- What kind of help?
- Who do you think might be able to help you?

1. If you were to be involved in a project to help other medical teachers in a similar situation as yourself with challenges in their role, what support, help or training do you think you would need to do this?

**Possible prompts:**

- Can you imagine trying to help yourself and other medical teachers in a similar situation as yourself in regards to the challenges in their role? If you wanted to do this – what might stop you? What might help?

1. Do you have anything else you would like to say or share with me on the subject we have been talking about?
